# Achieving Ti-5Al-4Sn-2Zr-1Mo-0.25Si-1Nb Alloys with High Strength and Moderate Ductility through Selective Laser Melting

**DOI:** 10.3390/ma13235527

**Published:** 2020-12-03

**Authors:** Jiangtao Ran, Xiaojing Sun, Shiliang Wei, Zhuo Chen, Hong Zhao

**Affiliations:** 1 College of Mechanical and Electrical Engineering, Harbin Engineering University, Harbin 150001, China; Ranjiangtao_heu@163.com; 2 College of Materials Science and Chemical Engineering, Harbin Engineering University, Harbin 150001, China; sunxiaojing@hrbeu.edu.cn; 3 Mechanical &Power Engineering College, Harbin University of Science and Technology, Harbin 150001, China; weishiliang@hrbeu.edu.cn; 4 Aerospace Hiwing (Harbin) Titanium Industry Co., Ltd., Harbin 150001, China; cccccczhuo@163.com

**Keywords:** selective laser melting, TA32 titanium alloy, microstructure, micro-zone composition analysis, mechanical properties

## Abstract

Ti-5Al-4Sn-2Zr-1Mo-0.25Si-1Nb (TA32) titanium alloy is a kind of near α high temperature titanium alloy with great application prospects in aero-engine afterburners and cruise missiles. However, there are still few studies on the microstructure and mechanical properties of TA32 specimens produced by selective laser melting (SLM) technology. In this study, TA32 specimens with high strength (tensile strength of 1267 MPa) and moderate ductility (elongation after fracture of 8%) were obtained by selective laser melting. The effect of laser power on the microstructure and mechanical behavior was studied and the results demonstrated that the average grain size increases with increasing laser power from 200 W to 400 W. Micro-zone composition analysis was carried out by energy dispersion spectrum (EDS), showing that the Al concentration inner grains is higher than that near grain boundaries. Fracture analysis results demonstrated that the fracture mode of SLM TA32 specimens was cleavage fracture. The tensile strength of the specimens built with a laser power of 250 W at 500 °C, 550 °C and 600 °C was measured as 869 MPa, 819 MPa and 712 MPa, respectively.

## 1. Introduction

Additive manufacturing of titanium alloys has attracted an increasing amount of attention because there is still a big challenge for the fabrication of titanium alloy parts with traditional methods, especially when complex geometries are taken into consideration. Compared to traditional manufacturing methods, the most significant advantage of additive manufacturing (AM) is its freeform fabrication capability of complex parts directly from feedstock materials, leading to low material waste and high liberalization [1,2,3,4].

Selective laser melting (SLM) is one promising additive manufacturing process and this technique can manufacture near net-shape metal parts as high as 99.9% relative density [5]. The microstructure of titanium alloys produced by selective laser melting are carried out by numerous literatures. Fine acicular α’ martensite was found to exist in the as-built SLM TC4 alloys due to the extremely high cooling rate [6,7,8,9,10,11,12,13]. The width of these acicular α′ needles varied from microns in the case of primary α′ down to nanometers for quaternary α′ [7]. These α′ needles can be categorized into four subgroups: primary martensite phase, secondary martensite phase, tertiary martensite phase and quaternary martensite phase [14,15]. Columnar prior β grains were also found to span across a few deposition layers and grow epitaxially parallel to the build orientation [7,12]. In addition, the microstructure of Ti-5Al-2.5Sn [16,17,18], Ti-22Al-25Nb [19,20], Ti-40Al-9V-0.5Y [21], Ti-6Al-7Nb [22], NiTi [23,24] were also discussed.

The mechanical properties of SLM titanium alloys varied greatly even if the same powder stock was adopted to build samples. According to the existing literatures, the ultimate tensile strength of SLM Ti-6Al-4V at room temperature varied from 950 MPa to 1421 MPa [25,26,27,28], while the elongation after fracture varied from 1.4 to 18.5% [25,26,27,28,29]. In general, titanium alloys produced by selective laser melting showed a characteristic of high strength and low ductility and an increasing number of research has been carried out to improve the ductility of SLM materials while maintaining high strength. An ultrafine α + β microstructure with high strength (>1100 MPa) and high ductility (11.4% elongation to failure) was obtained through the optimization of energy density [30]. The fabrication of strong and ductile Ti-6Al-4V alloys was also achieved when the optimal parameters was adopted, and the tensile strength was as high as 1170 MPa while the elongation after fracture also reached 10% [8]. Nevertheless, achieving high strength and excellent ductility is still challenging for SLM materials. Taking this into consideration, various additional elements were added to the Ti-based alloys. The influence of different mass fraction of Nb on the microstructure and properties of Ti-Al-Mn-Nb titanium alloys were investigated, showing that a better combination of hardness (HV 2000), strength (1390 MPa) and plastic deformation (24.5%) can be obtained when adding 7.0% (mass fraction) Nb [31]. Different mass fraction of B additions was added to Ti-6Al-4V and the microstructures and melt pool geometry features were researched, the results demonstrated that Ti6Al4V-xB with 2–5 wt % B as a promising composition range for SLM processing [32]. The effects of minor B_4_C addition on microstructure evolution, hardness, compressive properties and fracture mechanisms of the composites were systematically investigated, the results demonstrated that the Vickers hardness of the SLM nanocomposites increased 45% while the ultimate compressive strength increased 26% compared to the Ti-6Al-4V alloy [33]. Effect of nano-yttria stabilized zirconia (nYSZ) addition on the microstructure and the corresponding mechanical performance of SLM Ti-6Al-4V specimens was carried out, showing that the microhardness of Ti-6Al-4V was increased from 340 HV to 511 HV after adding nYSZ, with a maximum yield stress of 1302 MPa and compressive strength of 1751 MPa [34]. TiB_2_/Ti6Al4V multi-materials were manufactured by selective laser melting and the results showed that a variation nano-hardness was developed with chemical compositions at the interfacial regions [35].

As a new type of near α titanium alloy, Ti-5Al-4Sn-2Zr-1Mo-0.25Si-1Nb titanium alloy (TA32) has the advantages of high specific strength, creep resistance and good weldability, with an excellent combination of thermal strength and thermal stability at 550 °C, which is a kind of high temperature titanium alloy with great application potential in aero-engine afterburner and cruise missile. In this paper, TA32 titanium alloy with high strength (1280 MPa) and high ductility (8.5%) was obtained by selective laser melting. The microstructure of SLM TA32 alloys was researched by using optical microscope, scanning electron microscope and X-ray diffraction. The mechanical properties both at ambient temperature and elevated temperature were also characterized.

## 2. Materials and Methods

### 2.1. Materials and Processing Conditions

**Figure 1:**
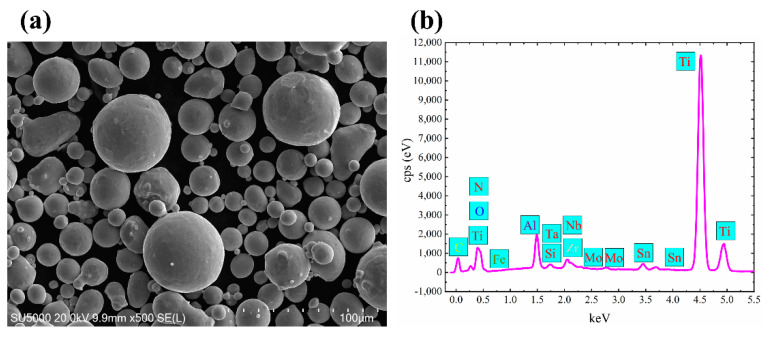
Properties of as-receive Ti-5Al-4Sn-2Zr-1Mo-0.25Si-1Nb titanium alloy (TA32) powders: (**a**) SEM morphology of TA32 powders; (**b**) the corresponding EDS results of elements.

The powders used in this research were gas atomized TA32 spherical powders provided by Aerospace Hiwing (Harbin) Titanium Industry Co., Ltd. (Harbin, China). As it is shown in Figure 1, its nominal diameter is between 21.9 and 49.2 μm. While the chemical compositions of as-received powders were as follows: Al:5.29; Sn:3.40; Zr:2.98; Mo:0.57; Si:0.28; Nb:0.41; Ta:0.40; Fe:0.026; C:0.0080; N:0.016; O:0.13; H:0.0017 and Ti balance.

From Figure 1, it is evident that the morphology of these powders appears to be spherical or nearly spherical, with some smaller powder particles attached to bigger powders.

All these specimens were built with the FS271M machine (Hunan Farsoon High-Technology Co., Ltd., Changsha, China) equipped with a continuous wavelength Ytterbium fiber laser with maximum beam spot size of 200 μm, and maximum power of 500 W. The employed scanning strategy in this study was round-trip scanning and the rotation angle between two consecutive layers was 67°. During the manufacturing process, the protective gas in the build chamber was ultra-high purity argon, so as to keep the oxygen concentration less than 0.02%. The build was started on the moment when a preheat temperature of 200 °C was achieved. All of these specimens were built in one batch and subjected to annealing of 800 °C for 2 h under the vacuum condition followed by furnace cooling to eliminate the residual stress generated during the building process and improve the ductility of this material.

### 2.2. Sample Location and Preparation

**Figure 2:**
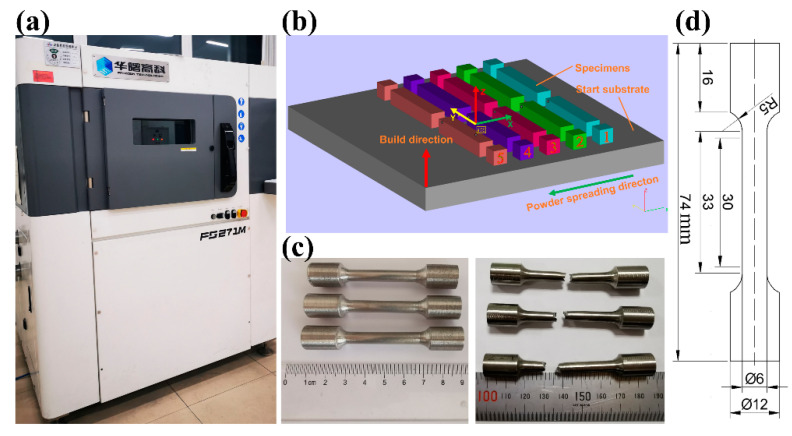
A photograph showing the manufacture of TA32 specimens: (**a**) FS271M machine; (**b**) layout of specimens on the base plate; (**c**) the machined specimens before and after fracture; (**d**) geometry dimension of tensile specimens (all units are mm).

The location and orientation of the TA32 specimens on the build platform are shown in Figure 2 and the specimens with the same color were built with the same process parameters. For each set of process parameters, two small cubic specimens with a geometry dimension of 12 mm × 12 mm × 12 mm and two rectangular tensile specimens with a geometry dimension of 15 mm × 84 mm × 15 mm were built. The process parameters used to manufacture these specimens are shown in Table 1.

All these specimens were built with the same scan speed, scan line hatch spacing and powder layer thickness, but with a different laser power, as detailed in Table 2. Before this experiment was carried out, a series of small cubic TA32 specimens (12 mm × 12 mm × 12 mm) with different process parameters were built by selective laser melting to investigate the influence of process parameters on the porosity and Vickers hardness of SLM TA32 specimens, and the results showed that laser power had the greatest influence on the porosity and Vickers hardness. What is more, the porosity of samples built with a scan speed of 1000 mm/s or a hatch spacing of 0.12 mm was less than those built with other scan speeds or hatch spacings. As a result, the scan speed and hatch spacing were kept as a constant in this study, so as to better investigate the effect of laser power on the microstructure and mechanical properties of SLM TA32 samples.

### 2.3. Characterization Methodology

#### 2.3.1. Microstructure Ans Porosity Characterization

The geometry dimension of specimens used for microstructure analysis is 12 mm × 12 mm × 12 mm, which were directly built on the substrate. After these samples were separated from the substrate, the top surface of these samples was mechanically ground and then polished with colloidal suspension (Struers 50 nm). Kroll’s reagent (a solution containing 10 mL HF, 30 mL HNO_3_ and 100 mL distilled water) was used to etch these samples for about 15 s. Leica DMI 5000 M (Shenyang Changxiao Instrument Co., Ltd, Shenyang, China) metallographic microscope and FE-SEM SU5000 (Hitachi High Technologies corporation, Shanghai, China) scanning electron microscope were used to analyze and characterize the microstructure of TA32 specimens.

Phase characterization was carried out using a Bruker D8 Advance X-ray diffractometer (Bruker Corporation, Billerica, MA, USA), with a diffraction angle 2θ, from 10° to 90° and a step size 0.02°. A tube source voltage of 40 kV and current of 40 mA were used. All XRD patterns were collected using Cu Kα1 radiation at 1.5406 nm wavelength.

The Archimedes method [36], the computed microtomography method [37] as well as the micrographic observations method can be used for evaluating porosity or relative density. While in this study, the micrographic observations method was adopted to measure the porosity of these SLM TA32 specimens. The polished surface of these samples was observed at a magnification of 50× with DMI 5000 M metallographic microscope. At this magnification, the area of each micrograph was 2.45 mm × 1.82 mm. A total of 30 micrographs were taken to cover the entire surface as much as possible for each sample. Then all these micrographs were processed by image processing software (Image-J, V1.8.0.112, National Institutes of Health, Bethesda, MD, USA) with the same threshold. The ratio of the pixel of the pores to the total pixel of one micrograph is defined as the porosity.

#### 2.3.2. Microhardness

TMVS-1S (Future technology, Beijing, China) digital microhardness tester was used to measure the Vickers hardness of TA32 specimens. The loading force was 9.8 N and the dwell time was 15 s in the measuring process. Five points were randomly measured on the top surface, and the average value was taken as the Vickers hardness of each sample.

#### 2.3.3. Tensile Testing

For tensile tests at ambient temperature, a mill and lath were adopted to machine these specimens into tensile specimens. The gauge diameter and gauge length of these tensile specimens was 6 mm and 30 mm, respectively, which was illustrated in Figure 2. The Instron 8862 high-precision fatigue testing machine (Instron, Boston, MA, USA) was used to measure the mechanical properties of TA32 specimens with a strain rate of 1 × 10^−3^ s^−1^. Two samples were measured for each set of process parameters, taking the average value as the actual value.

For tensile tests at elevated temperature (500 °C, 550 °C, 600 °C), a CMT5105 (Measure Test Simulate Technology Co., Ltd, Beijing, China) high and low temperature electronic universal testing machine was adopted, with a strain rate of 0.00025 s^−1^. Cylindrical specimens with threads at both ends were used to evaluate the mechanical properties of SLM TA32 specimens at elevated temperature. The gauge diameter was 5 mm while the gauge length was 40 mm. Only these specimens built with a laser of 250 W were tested for the reason that these specimens have a better combination of strength and ductility at ambient temperature. For each test temperature, two specimens were measured to obtain the average value.

## 3. Results and Discussion

### 3.1. Microstructure of SLM TA32 Titanium Alloy

**Figure 3:**
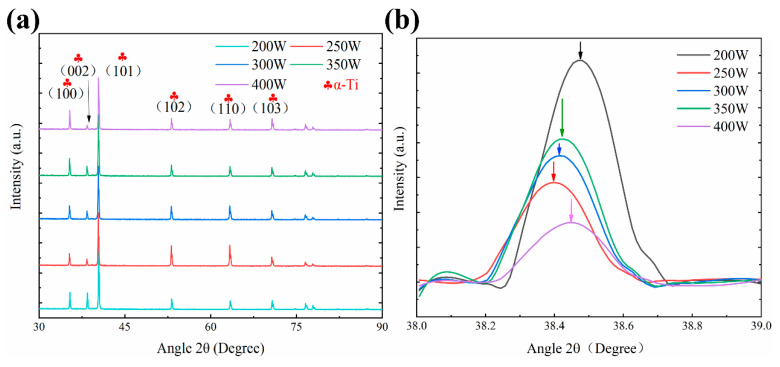
XRD patterns of selective laser melting (SLM) TA32 specimens with different laser power: (**a**) XRD patterns of SLM formed samples; (**b**) local XRD patterns of SLM formed samples (38°–39°).

Figure 3 shows the XRD patterns of TA32 samples formed with different laser powers. The diffraction peaks of all samples have the characteristics of hexagonal close packed (HCP) phase, which is composed of α phase and α’ martensite phase. It is more favorable for the formation of martensite phase due to the extremely high cooling rate of selective laser melting. As a result, the hexagonal close-packed phase is considered to be α’ martensite phase. When the laser power increases from 250 W to 400 W, the peak value of α phase shifts to the right. According to Bragg’s law, when the angle of θ increases, d will decrease, indicating that the spacing between crystal planes decreases. Al is dissolved in Ti-matrix to form solid solution and the atomic radius of aluminum is smaller than that of titanium. The solid solution of aluminum in titanium will reduce the spacing between crystal planes; that is, the peak value shifts to the right. When the laser power is 200 W, the diffraction peak is steep and narrow, indicating that the grains are fine; when the laser power is 400 W, the diffraction peak is wide and slow, indicating that the grains are coarser.

**Figure 4:**
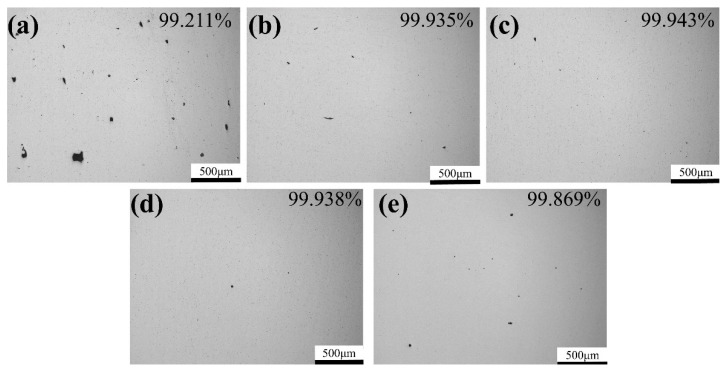
Influence of laser power on the polished surface morphology of SLM TA32 specimens: (**a**) 200 W; (**b**) 250 W; (**c**) 300 W; (**d**) 350 W; (**e**) 400 W.

All these samples were ground and polished but not etched so as to observe the surface morphology and the results are shown in Figure 4. It is evident that there is a huge number of pores or build defects with a laser power of 200 W. When increasing laser power from 200 W to 350 W, the number and size of pores or lack of fusion defects show a decreasing tend. However, when a laser power of 400 W is adopted, the number of defects is increasing to some degree. The results show that pores or lack of fusion defects are easier to form with an extremely high or low laser power. On the one hand, when the laser power is too low, the energy input is insufficient to fully melt the powders. Under this circumstance, these powders will be partially melted or even not melted, leading to the formation of pores or build defects. On the other, when the laser power is too high, Al and other elements will vaporize and produce recoil pressure, resulting in the keyhole effect, which will generate pores after the solidification of molten pool. Therefore, in order to effectively reduce the number and size of defects in SLM TA32 samples, the laser power should be in the range of 250–350 W.

**Figure 5:**
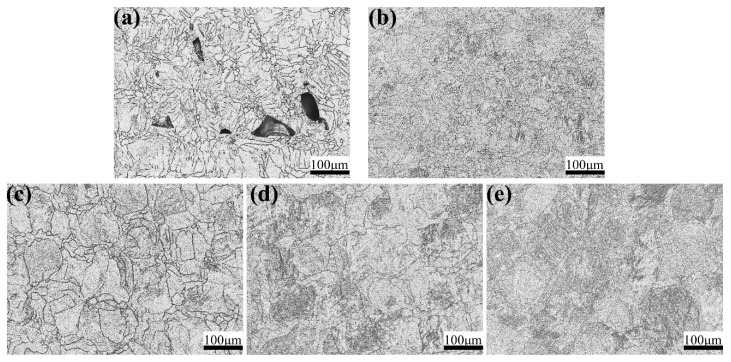
Effect of laser power on grain size of SLM TA32 specimens: (**a**) 200 W; (**b**) 250 W; (**c**) 300 W; (**d**) 350 W; (**e**) 400 W.

**Figure 6:**
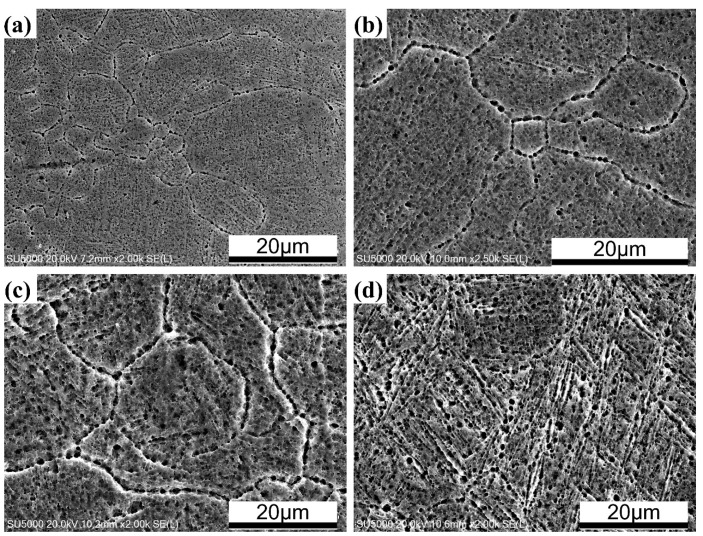
Scanning electron microscope photographs showing the various microstructures of TA32 specimens built with different laser powers: (**a**) 200 W; (**b**) 250 W; (**c**) 300 W; (**d**) 350 W.

Figure 5 and Figure 6 show the influence of laser power on the microstructure of SLM TA32 samples. When increasing laser power from 200 W to 400 W, the average grain size shows an increasing trend, which is shown in Figure 5. Lack of fusion defects are visible for TA32 specimens built with a laser power of 200 W, as is illustrated in Figure 5a. In addition, the microstructure of TA32 samples varies from cellular microstructure to dendrite microstructure, which is shown in Figure 6.

In order to evaluate the grain size quantitatively, the intercept procedure was used to measure the average grain size. First of all, ten micrographs were taken at the magnification of 200× for each sample. These ten micrographs were obtained from one plane but different positions of the top surface. Then a horizontal line was drawn to span across as many grains as possible on one micrograph and the length of the line was recorded. The number of grains covered by this horizontal line was also recorded. The average grain size is defined as the ratio between the length of this horizontal line and the number of grains covered by this line. Five lines were drawn for each micrograph and finally the average grain size of each sample was obtained. The measured average grain size increased from 12.4 μm to 62 μm when increasing laser power from 200 W to 400 W.

A lower laser power means a lower energy input, if the energy input is too low, lack of fusion defects will come into being. In addition, a lower energy input results in a higher cooling rate, which is more conducive to the nucleation of grains instead of crystal growth, leading to a rather smaller grain size. A higher energy input will be gained with a higher laser power, leading to a slower cooling rate, which is more beneficial to the crystal growth rather than crystal nucleation, resulting in the grain size becoming larger.

**Figure 7:**
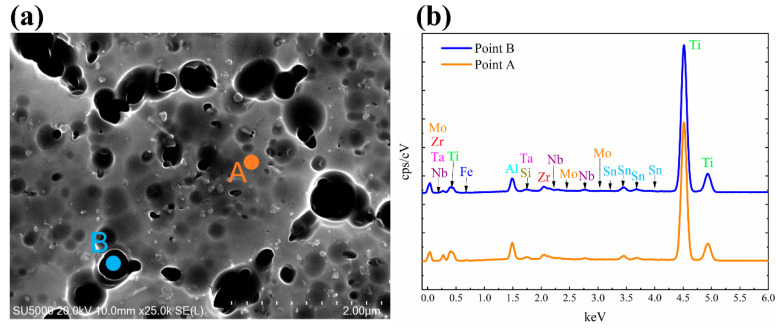
EDS analyses showing chemical composition variation in grain boundaries and grains of SLM TA32 specimen built with a laser power of 200 W: (**a**) selected position for EDS analysis and (**b**) the corresponding EDS spectrum.

**Figure 8:**
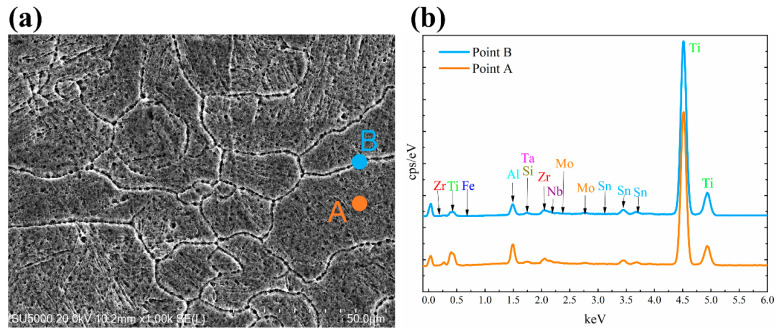
EDS analyses showing chemical composition variation in grain boundaries and grains of SLM TA32 specimen built with a laser power of 300 W: (**a**) selected position for EDS analysis and (**b**) the corresponding EDS spectrum.

**Figure 9:**
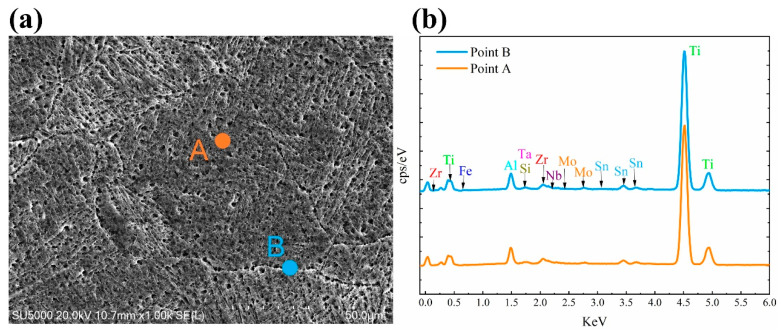
EDS analyses showing chemical composition variation in grain boundaries and grains of SLM TA32 specimen built with a laser power of 400 W: (**a**) selected position for EDS analysis and (**b**) the corresponding EDS spectrum.

Figure 7, Figure 8 and Figure 9 show the chemical composition variation in grain boundaries and grains of TA32 specimens built with a laser power of 200 W, 300 W, 400 W, respectively. The corresponding chemical element’s mass fraction is shown in Table 2. Point A is within the grains, while point B is at grain boundaries. It is evident that the chemical composition is different at grain boundaries and within grains.

For TA32 specimens built with three different laser powers, the concentration of Al in grain interiors is higher than that in grain boundaries. For specimen built with a laser power of 200 W, even if the grain size is only 2 μm the mass fraction of Al in grain interior is higher than that in grain boundary. For specimen built with a laser power of 300 W, the mass fraction of Al in grain interior is almost twice of that in grain boundary. However, for specimen built with a laser power of 400 W, the mass fraction of Al in grain interior is almost the same as that in grain boundary.

The maximum temperature of the molten pool is determined by laser power; thus, a higher laser power will lead to a higher temperature in molten pool. When a laser power of 200 W is adopted, the temperature in the molten pool is relatively lower. At this circumstance, the ability of atomic diffusion is weak to some degree. In addition, a lower laser power means a lower energy input, resulting in the rapid cooling and solidification of molten pool when laser beam moves away. Consequently, elements diffusion of Al is not sufficient, leading to a different Al concentration in grain interior and grain boundary, even if the grain size is rather small. When a laser power of 400 W is adopted, the temperature in molten is higher and the atomic diffusion ability is strong. Elements diffusion of Al is highly sufficient in such a situation, thus, Al concentration in grain interior and grain boundary is almost the same.

**Figure 10:**
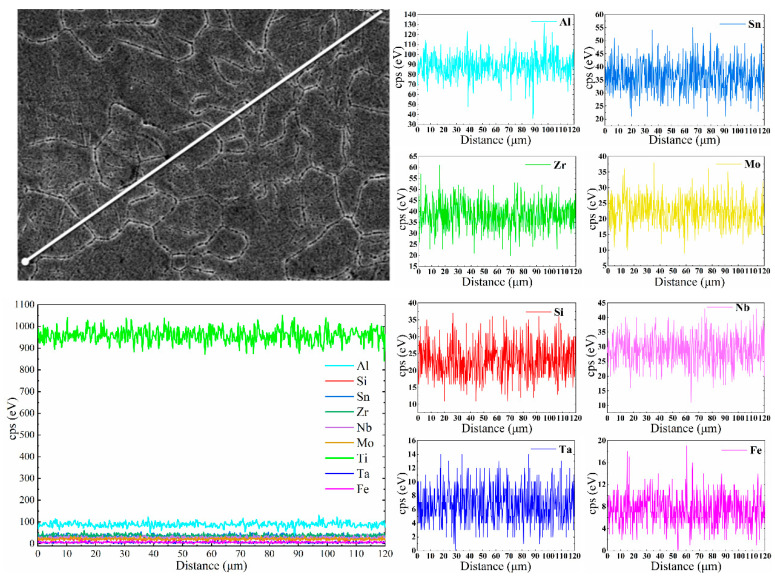
EDS line scan showing chemical composition variation along the scan line of SLM TA32 specimen built with a laser power of 250 W.

**Figure 11:**
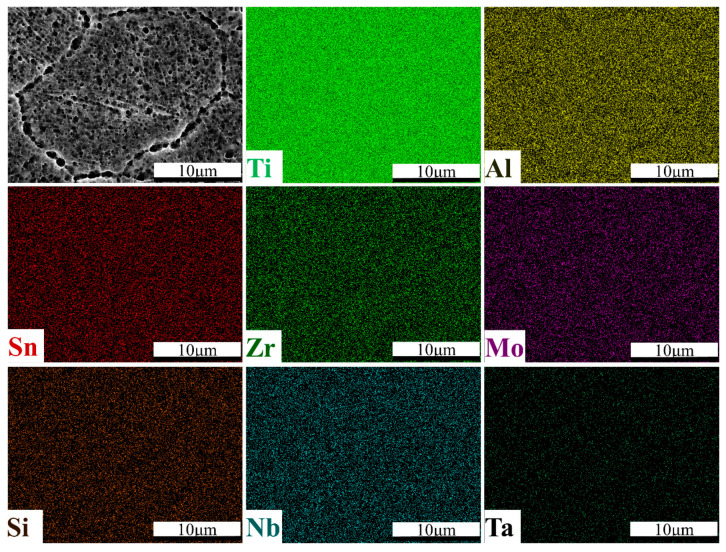
EDS maps showing the elements distribution in the measured area of SLM TA32 specimen built with a laser power of 250 W.

Figure 10 and Figure 11 show the EDS line scan and area scan results of TA32 specimen built with a laser power of 250 W, respectively. It is evident from Figure 10 that chemical composition varies greatly along the scan line. Generally speaking, the content of Al in grain boundary is relatively lower, while the distribution of other elements is nearly uniform along the whole scan line. This phenomenon is also confirmed by EDS mappings, showing a lower content of Al at grain boundary.

As mentioned above, a relatively low laser power will cause the insufficient diffusion of Al, leading to a different Al concentration in grain interiors and grain boundary. To sum up, laser power is of great importance to the element’s diffusion of Al. A low laser power will lead to a rather lower Al concentration in grain boundary. A higher laser power makes the elements diffusion more sufficient; thus, Al concentration is almost the same in grain interiors and grain boundary.

### 3.2. Mechanical Properties of SLM TA32 Specimens

#### 3.2.1. Mechanical Properties at Ambient Temperature

**Figure 12:**
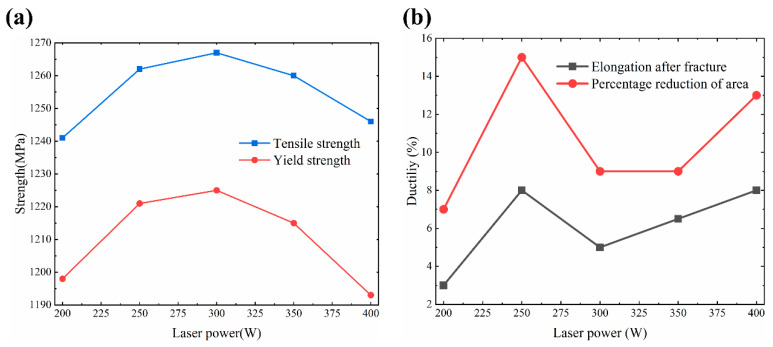
Effect of laser power on the strength and ductility of SLM TA32 specimens at ambient temperature:(**a**) Effect of laser power on the tensile strength and yield strength of TA32 specimens; (**b**) Effect of laser power on the ductility of TA32 specimens

High strength and high ductility are always the eternal pursuit of materials built by selective laser melting technology. Nevertheless, due to the formation of martensite phase, SLM materials have a characteristic of high strength but low ductility. The effect of laser power on the strength and ductility of SLM TA32 specimens is shown in Figure 12. High strength is obtained for specimens built with different laser powers; however, ductility varies greatly. As for strength, tensile strength and yield strength increase first and then decrease with increasing laser power. The tensile strength of specimens built with a laser power of 300 W is the highest, which is measured as 1267 MPa. As for ductility, no obvious trend is found with increasing laser power, but the elongation after fracture of specimen built with a laser power of 250 W is the highest, which is measured as 8%, showing a tensile strength of 1262 MPa. A tensile strength of 1262 MPa is higher than that of Ti-6Al-4V [26,28,29,38,39,40,41,42], comparable to that of Ti-6Al-4V [27,43], lower than that of Ti-6Al-4V [25]. To our best knowledge, the highest tensile strength of Ti-6Al-4V is 1420 MPa [25]. An elongation of 8% is higher than that of Ti-6Al-4V [25,27,40,43], comparable to that of Ti-6Al-4V [26,28,41], but lower than that of Ti-6Al-4V [29,38]. By adopting hot isostatic pressing (HIP) process, the elongation of SLM Ti-6Al-4V can be as high as 19.4% [29]. Methods to further improve the ductility while maintaining the high strength of TA32 specimens will be the focus of our future research.

Generally speaking, materials with finer grains have better strength and ductility; however, the effect of defects on strength and ductility cannot be neglected. Build defects have a detrimental impact on the mechanical properties of parts fabricated by additive manufacturing. When these parts are subjected to cyclic loads, build defects can develop into crack nucleation site, leading to premature failure of these parts [44]. Build defects, such as large continuous pores, significantly deteriorate the metallurgical bonding between adjacent melts, leading to easier cracking in the process of tensile tests [20]. As a result, the tensile properties of specimens with large defects are worsened. In this study, although the average grain size of the samples built with a laser power of 200 W is the smallest, a huge number of defects with large size are also detected within these specimens. This is the reason for the lower strength and ductility, especially for elongation after fracture, which is measured as 3%.

**Figure 13:**
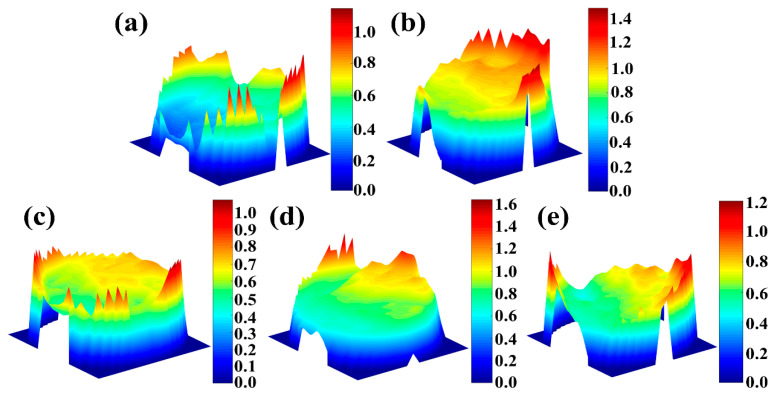
Effect of laser power on the 3D morphologies of the fracture of TA32 specimens: (**a**) 200 W; (**b**) 250 W; (**c**) 300 W; (**d**) 350 W; (**e**) 400 W.

Figure 13 shows the 3D fracture morphologies of TA32 specimens built with different laser powers. It is evident that laser power has a significant influence on the fracture morphologies. For specimens built with a laser power of 200 W, the fracture surface is relatively flat overall, showing some sharp protrusions at the edge of the fracture. For specimens built with a higher laser power (250 W–400 W), a shear slipping fracture is visible.

**Figure 14:**
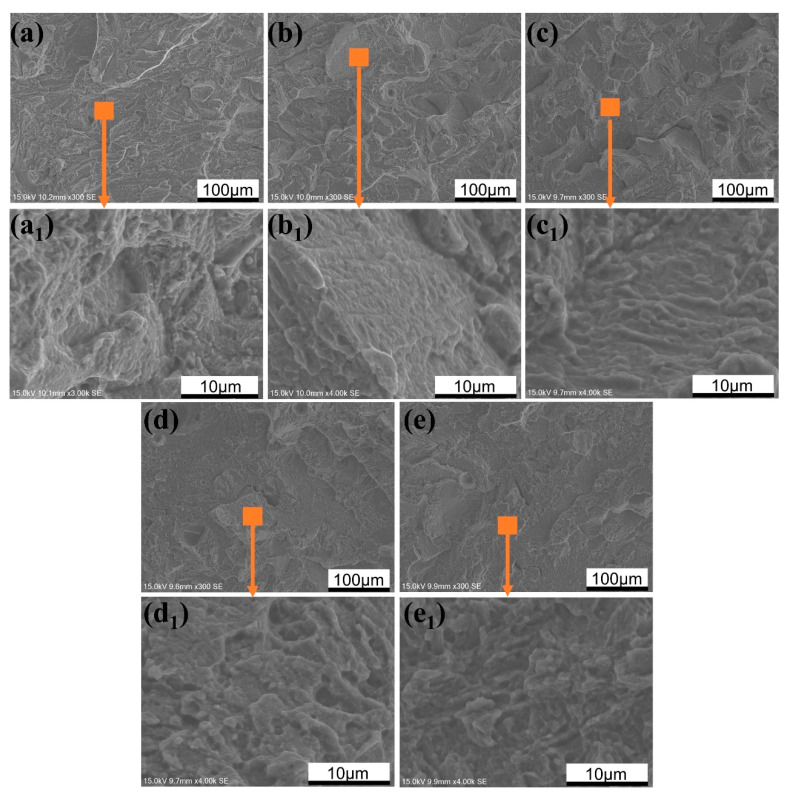
Effect of laser power on the microscopic fracture morphology of TA32 specimens: (**a**,**a1**) 200 W; (**b**,**b1**) 250 W; (**c**,**c1**) 300 W; (**d**,**d1**) 350 W; (**e**,**e1**) 400 W.

Figure 14 shows the microscopic fracture morphologies of SLM TA32 specimens built with different laser powers. Partially melted metal powders are observed on the fracture surfaces of the specimens manufactured with a laser power of 200 and 250 W, which is shown in Figure 14a,b. River-like patterns are observed on the fracture surfaces of all these specimens, which is shown in Figure 14a–e. No visible dimples are observed in SEM photographs at higher magnification for all the specimens. Based on the above analysis results, it can be concluded that the fracture mode of TA32 specimens is cleavage fracture. Generally speaking, the ductility of materials is poor when cleavage fracture occurs, and this is the reason why the elongation of all specimens is less than 10%.

#### 3.2.2. Mechanical Properties at Elevated Temperature

**Figure 15:**
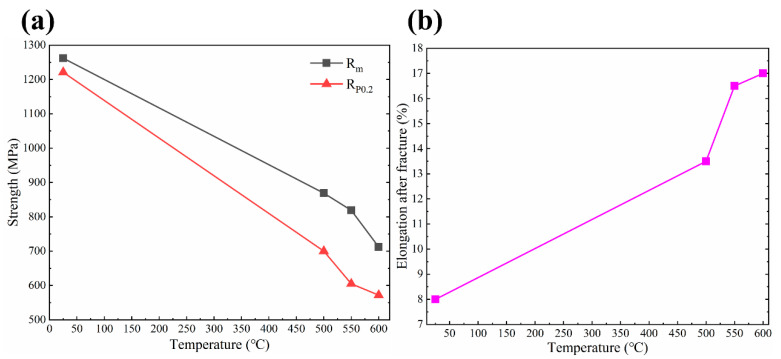
Effect of testing temperature on the mechanical properties of TA32 specimen: (**a**) Effect of testing temperature on the tensile strength (Rm)and yield strength (Rp0.2) of TA32 specimens; (**b**) Effect of testing temperature on the elongation of TA32 specimens.

TA32 titanium alloy has attracted more and more attention because of its high thermal strength and thermal stability at 550 °C. As a result, the tensile strength at elevated temperature is also characterized. Figure 15 shows the evolution law of strength and ductility with an increase of temperature. It is evident that both tensile strength and yield strength decrease with the increase of temperature. When the testing temperature increases from ambient temperature to 500 °C, the strength decreases rapidly, from 1262 MPa to 869 MPa. The decrease of strength gradually slows down with increasing temperature from 500 °C to 600 °C. The tensile strength of TA32 specimen at 550 °C is measured as 819 MPa, which is close to the strength of Ti-6Al-4V at ambient temperature, indicating that this material has high strength at elevated temperature. As for ductility, the elongation after fracture increases with increasing testing temperature. An elongation of 17% is gained with a test temperature of 600 °C.

With the increase of temperature, the diffusion ability of atoms is enhanced, leading to the formation of vacancies, which will gradually evolve into defects and reduce the properties of materials. What is more, the resistance of dislocation movement is smaller at high temperature, dislocations move more easily, making the material strength descend. A possible explanation for the higher elongation at elevated temperature is that critical shear stress decreases with the increase of temperature, leading to the increase of slip systems.

### 3.3. Microhardness Measurements

**Figure 16:**
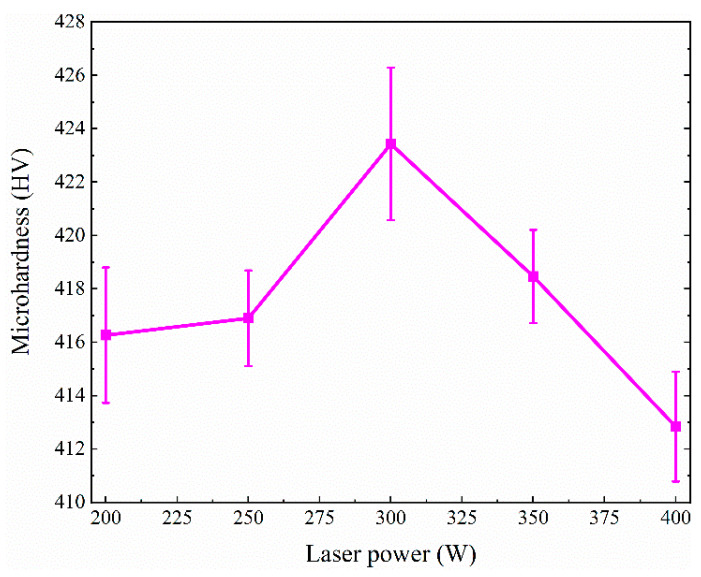
Effect of laser power on the microhardness of SLM TA32 specimens.

The influence of laser power on Vickers hardness of SLM TA32 specimens is shown in Figure 16. It is evident that the Vickers hardness of TA32 specimens increases first and then decreases with increasing laser power, which is consistent with the evolution law of material strength, indicating that there is a positive correlation between the strength and hardness of this material. When laser power was increased from 200 W to 300 W, the Vickers hardness of TA32 specimens increased from 416 HV to 423 HV. As laser power increased from 300 W to 400 W, the Vickers hardness of TA32 specimens decreased from 423 HV to 412 HV.

The Vickers hardness of these specimens varies from 410 HV to 425 HV. This is higher than the value reported in Refs. [8,9,45,46] for SLM Ti-6Al-4V, lower than that of Ti-40Al-9V-0.5Y alloy [21], as well as γ-TiAl-based alloys with Nb additions [31]. Ismaeel et al. [31] have demonstrated that the γ-TiAl-based alloy with a 7.0 at.% Nb addition has a microhardness of HV 2000, owing to grain refinement and enhanced solution strengthening. The reason why the Vickers hardness of TA32 specimens is higher than that of Ti-6Al-4V should be attributed to the addition of Nb.

## 4. Conclusions

The effect of laser power on the microstructure and mechanical properties of TA32 titanium alloy produced by selective laser melting was studied in this study. Based on microstructure analysis, energy spectrum analysis, fracture analysis and Vickers hardness measurement as well as tensile tests at ambient and elevated temperatures, the following conclusions are drawn:

(1) Laser power has a significant effect on the microstructure of SLM TA32 specimens in the way that the average grain size increases from 12.4 μm to 62 μm when increasing laser power from 200 W to 400 W. When laser power increases, the microstructure of TA32 specimens varies from cellar microstructure to dendrite microstructure.
(2) When increasing laser power, the tensile strength and yield strength of SLM TA32 specimens increase first and then decrease. The maximum value of TA32 specimen is measured as 1225 MPa with a laser power of 300 W. While increasing laser power, the ductility of TA32 specimens has no obvious developing trend. The strength and ductility of TA32 specimens are significantly affected by build defects. Specimens built with a laser power of 200 W show a lower strength and ductility due to the occurrence of a huge number of defects with large size. Fracture analysis results demonstrated that the fracture mode of SLM TA32 specimens is cleavage fracture.
(3) Al concentration inner grains is higher than that near grain boundaries for specimens built with different laser powers; however, the concentration of Al element is nearly the same in grains and grain boundaries when a laser power of 400 W was adopted, indicating that the elements diffusion is more sufficient with a relatively higher laser power.
(4) The tensile strength of the specimens built with a laser power of 250 W at 500 °C, 550 °C and 600 °C was measured as 869 MPa, 819 MPa and 712 MPa, respectively. The tensile strength of TA32 specimens at 550 °C is close to that of Ti-6Al-4V alloy at ambient temperature, which demonstrates that SLM TA32 specimens have an excellent high temperature mechanical property.

## Figures and Tables

**Table 1:** Process parameters used for producing TA32 specimens through selective laser melting.

Sample ID	1	2	3	4	5
Laser power/W	200	250	300	350	400
Scan speed/(mm/s)	1000				
Scan line hatch spacing/mm	0.12				
Thickness of powder layers/mm	0.06				

**Table 2:** Mass fraction of various elements in grain boundaries and grains of TA32 specimens built with a laser power of 200 W, 300 W, 400 W.

Laser Power/W	Position	Elements	Al	Si	Ti	Fe	Zr	Nb	Mo	Sn	Ta
200	A	wt.%	5.03	0.41	86.27	0.00	3.08	0.54	0.62	3.76	0.30
200	B	wt.%	3.92	0.23	87.58	0.00	2.86	0.62	0.67	3.52	0.60
300	A	wt.%	5.44	0.35	86.10	0.04	2.94	0.68	0.51	3.74	0.21
300	B	wt.%	2.78	0.32	90.01	0.09	2.29	0.35	0.45	3.69	0.01
400	A	wt.%	4.97	0.31	87.36	0.03	2.99	0.45	0.44	3.43	0.03
400	B	wt.%	4.84	0.29	86.96	0.00	2.86	0.61	0.57	3.73	0.14
